# S100A4 facilitates influenza A virus replication by interacting with M2 in human alveolar epithelial cells

**DOI:** 10.3389/fmicb.2026.2011614

**Published:** 2026-10-29

**Authors:** Li Wu, Yingying Tao, Jianing Li, Rui Yang, Jiasheng Song, Jianhong Shu, Yulong He, Huapeng Feng

**Affiliations:** 1 Department of Biology, College of Life Sciences, China Jiliang University, Hangzhou, China; 2 Department of Biopharmacy, College of Life Sciences and Medicine, Zhejiang Sci-Tech University, Hangzhou, China; 3 Zhejiang Difference Biotechnology Co., Ltd, Hangzhou, China

**Keywords:** host factor, influenza A virus, M2 protein, replication, S100A4

## Abstract

Influenza A virus (IAV) replication depends on host cells and is regulated by numerous host factors. Therefore, investigating and understanding the role of these host factors is a significant topic. In this study, we found the IAV replication was attenuated after host factor S100 calcium-binding protein A4 (S100A4) was knocked down with RNAi or knockout with CRISPR/Cas9 gene-editing technology. Conversely, IAV replication was enhanced upon the overexpression in A549 cells. The interaction between S100A4 and the IAV M2 protein was confirmed by co-immunoprecipitation. Meanwhile, confocal microscopy experiment demonstrated that the viral M2 protein co-localized with endogenous S100A4 in IAV-infected A549 cells. Truncation analysis confirmed that amino acids 45–62 of the M2 protein constitute the main interaction region with S100A4. Further study showed that viral entry was reduced after S100A4 deletion. And the polymerase activity was significantly decreased in S100A4-knockout A549 cells compared to that in wild-type A549 cells. The S100A4 overexpression resulted in the downregulation of IFN-β, while the mRNA levels of the interferon-stimulated genes ISG15 and BST2 were also significantly reduced. Therefore, the host factor S100A4 was a positive modulator of IAV replication and was involved in the viral entry, polymerase activity maintenance and innate immune regulation. Collectively, our findings shed new light on the molecular mechanisms governing IAV infection and suggest a previously underexplored avenue for antiviral therapeutic intervention.

## 1 Introduction

Influenza A virus (IAV) is an enveloped RNA virus within the family Orthomyxoviridae. Its genetic material is organized into eight distinct RNA segments, forming a segmented genome of approximately 13 kb. IAV can infect wild birds, poultry, pigs, horses, and various other mammals, including seal, dairy cattle, and humans (Peacock et al., 2025). Through continuous genetic variation involving antigenic drift as well as reassortment associated with antigenic shift, IAV has generated viruses capable of causing widespread human disease. Major pandemic events occurred in 1918 (H1N1), 1957 (H2N2), 1968 (H3N2), and 2009 (H1N1) (Krammer et al., 2025). Human infection is not restricted to seasonal influenza viruses; zoonotic transmission has also been documented for a range of avian influenza subtypes, such as H3N8, H5N1, H5N5, H5N6, H5N8, H7N7, H7N9, H9N2, H10N3, and H10N8 (Yang et al., 2022; Kang et al., 2024; World Health Organization, 2026). Among them, the H7N9 subtype avian influenza virus has infected 1,568 humans and caused 616 deaths in China, while Clade 2.3.4.4b H5N1 avian influenza virus circulated in dairy cattle in the USA and infected 71 humans with 2 deaths (Centers for Disease Control and Prevention, 2026; World Health Organization, 2026). Current approaches to limiting the burden of influenza largely involve the use of vaccines together with antiviral therapeutics. For avian influenza viruses, no approved vaccine for human use is currently available in China. For seasonal influenza viruses, the efficacy of seasonal influenza vaccine decreases due to the antigenic mismatch between the influenza vaccine strain and circulation since the influenza virus mutates rapidly. The principal categories of licensed antiviral agents for influenza consist of M2 ion channel blockers, represented by amantadine, along with neuraminidase (NA) inhibitor (oseltamivir) and PA endonuclease inhibitor (baloxavir marboxil). However, the antiviral-resistant strains emerge soon after clinical use due to the rapid evolution of IAVs, including the S31N mutation in Matrix protein 2 (M2) confers resistance to amantadine, H275Y/R292K mutations in the NA protein confer resistance to oseltamivir and the PA I38T mutation confers resistance to baloxavir marboxil (Patel et al., 2026). Current anti-IAV drugs target viral proteins. The IAV life cycle depends on host cells, and a variety of host factors are involved in IAV replication (Jiang et al., 2023). Therefore, the discovery and characterization of novel host factors will provide new targets for the development of anti-influenza therapeutics.

Functioning as a surface ion channel, the M2 protein of IAV critically contributes to viral replication. Typically composed of 97 amino acid residues, M2 contains three major regions distributed along its sequence, with residues 1–24 constituting the ectodomain, residues 25–43 forming the transmembrane segment, and residues 44–97 corresponding to the C-terminal domain. The CTD itself harbors both functional motifs and regions that mediate protein-protein interactions, The membrane-embedded segment mediates proton transport in response to changes in pH, with His37 detecting proton availability and Trp41 regulating the transition between the conductive and nonconductive states (Hong and DeGrado, 2012; Manzoor et al., 2017; Kumar and Sakharam, 2024). Following endocytic entry, M2 contributes to the fusion event between the viral membrane and the surrounding endosomal membrane, and also contributes to the fission and separation of progeny virions from the host plasma membrane during egress, while simultaneously accommodating the pharmacologic target of amantadine (Chen et al., 2008). To date, several host factors that interact with the M2 protein have been identified. TRAPPC6A & TRAPPC6AΔ interacting with M2, mediated by a conserved leucine (L96) in M2, restricts M2 trafficking to the plasma membrane, favoring viral replication (Zhu et al., 2017). RAB33B contributes to the intracellular trafficking of M2 toward the cell surface and enhances IAV replication by regulating autophagy (Ye et al., 2025). LC3 interacts with M2 and caspase cleavage of M2 results in the disruption of the M2-LC3 interaction and affects viral filament formation and virion production (Figueras-Novoa et al., 2025). YWHAG negatively regulates the replication of IAV by suppressing the release of viral M2 and impairing virus budding (Mao et al., 2022). PEX19 interacts with M2 by its C-terminus and inhibits the replication of IAV. However, M2 could partially reduce this restriction by disrupting the peroxisome function (Liu et al., 2024). UBR4 is a proviral factor that interacts directly with the M2 protein and plays a critical role in the apical transport of viral proteins to facilitate the budding of IAV (Tripathi et al., 2015). Together, host factors interacting with influenza virus M2 serve as either proviral accomplices that promote trafficking, replication, and budding, or as restriction factors that suppress these processes, reflecting the complex interplay between the virus and its host.

S100A4, also known as fibroblast-specific protein 1 (FSP1), Mts1, or metastasin, is a calcium-binding member of the S100 protein family. This small protein composed of approximately 101 amino acids forms an anti-parallel homodimer, with each monomer containing two EF-hand motifs that undergo conformational rearrangement upon Ca^2^^+^ binding (Donato et al., 2013; Tao et al., 2026). Although S100A4 has been extensively investigated in tumor metastasis, fibrosis, and inflammatory disorders, accumulating evidence indicates that it also participates in host–virus interactions and antiviral immunity (Wong et al., 2025; Tao et al., 2026). Its biological functions are strongly influenced by its intracellular and extracellular localization. Intracellular S100A4 interacts with non-muscle myosin IIA and other cytoskeletal components, thereby regulating cytoskeletal organization and cell motility (Dulyaninova et al., 2005). Extracellularly, S100A4 can act as a damage-associated molecular pattern (DAMP) and stimulate inflammatory signaling through receptors including RAGE and TLR4. Increasing evidence has linked S100A4 to viral pathogenesis. During Zika virus infection, S100A4-positive macrophages facilitate viral invasion and persistence in the testes by increasing blood–testis barrier permeability and establishing an immune-permissive microenvironment that limits effective antiviral immunity (Yang et al., 2020). In addition, S100A4 has been implicated in the regulation of adaptive immune responses, while exogenous S100A4 can enhance mucosal antibody production and pulmonary CD8^+^ T-cell responses, highlighting its context-dependent immunomodulatory activity during viral infection (Sen Chaudhuri et al., 2022).

Despite these observations, the role of S100A4 in influenza virus infection remains largely unexplored. A549 cells, a human lung adenocarcinoma-derived cell line, are widely used as an *in vitro* model for investigating influenza A virus (IAV) replication and virus–host interactions (Zhou et al., 2011; Ujie et al., 2019). However, whether S100A4 influences IAV replication in human lung-derived cells has not been established. In the present study, we demonstrate that S100A4 promotes IAV replication in A549 cells and further investigate the molecular basis underlying this proviral effect. Our findings identify S100A4 as a previously unrecognized host factor that facilitates influenza virus replication and provide new insights into the contribution of S100A4 to influenza virus–host interactions in human lung epithelial cells.

## 2 Materials and methods

### 2.1 Cells and viruses

A549 cells (ATCC, CCL-185), derived from human lung adenocarcinoma epithelium, were maintained in Ham's F-12K medium supplemented with 10% FBS and 1% penicillin-streptomycin. Human embryonic kidney 293T cells (ATCC, CRL-3216) were grown in DMEM (Gibco) containing 10% FBS and 1% penicillin-streptomycin. MDCK cells (ATCC, CCL-34) were propagated in DMEM supplemented with 5% FBS and 1% penicillin-streptomycin. All cell cultures were maintained at 37 °C in a humidified atmosphere containing 5% CO_2_. Using reverse genetics, infectious A/WSN/1933 (H1N1; WSN) virus was rescued and subsequently amplified in MDCK cells under 37 °C conditions. Viral stocks were preserved at −80 °C until required for subsequent experiments (Neumann et al., 1999). To generate a recombinant virus carrying a luciferase reporter, the PB2 segment of the WSN strain was engineered such that its coding region was largely substituted with the *Renilla* luciferase gene, while retaining the essential 120-nucleotide packaging signal. This construct, designated PB2(120) Rluc(120), was assembled into the plasmid pPolI-WSN PB2-KO/Rluc. The corresponding virus, WSN PB2-KO/Rluc, was then recovered through reverse genetics by co-transfecting this plasmid together with the remaining seven pPolI-based transcription plasmids and four pCAGGS-based expression constructs encoding the WSN PB2, PB1, PA, and NP proteins with the procedure performed as reported previously (Ozawa et al., 2011). Propagation of the rescued virus was carried out in MDCK cells that stably express the PB2 protein (MDCK/PB2 cells). Separately, a mutant influenza virus termed PR8-NS1-R38A/K41A was produced by reverse genetics. This mutant was subsequently amplified in 10-day-old embryonated chicken eggs and kept at −80 °C until needed.

### 2.2 RNAi

Three pairs of small interfering RNAs (siRNAs) were designed based on the human S100A4 coding sequence published in GenBank (Accession No. NM_019554). The siRNAs and negative control (siNC) were synthesized by GenePharma (Shanghai, China). Table 1 presents the sequence data. A549 cells were subjected to transfection with three distinct siRNAs targeting S100A4, named as siS100A4-1#, siS100A4-2#, and siS100A4-3#. For RNAi experiments, cells were transfected in the presence of RNAiMAX (Thermo Fisher Scientific, Waltham, MA, USA). GenePharma non-targeting siRNA was included as the siNC control. After 48 h post-incubation, transfected cells were partially harvested, and the effectiveness of knockdown was examined at both the mRNA and protein levels using qRT-PCR and Western blotting, respectively.

**Table 1:** siRNA sequences.

siRNAs	Sequences(5^′^ → 3^′^)
siS100A4-1#	GUGACAAGUUCAAGCUCAAUU
	UUGAGCUUGAACUUGUCACUU
siS100A4-2#	GUGUCUUCCUGUCCUGCAUUU
	GTGTCTTCCTGTCCTGCATUU
siS100A4-3#	UGAGCAACUUGGACAGCAAUU
	UUGCUGUCCAAGUUGCUCAUU
siNC	GenePharma NC

### 2.3 Establishment of A549 cells overexpressing S100A4 or depleted S100A4

A549-derived cDNA served as the template for amplification of the human S100A4 open reading frame. During primer design, a Flag sequence was incorporated into the 3'end of the resulting amplicon. The PCR product was cloned into the A site of the pIRES-puromycin backbone, whereas the B site carries the puromycin-resistance element, generating the pIRES-S100A4-Flag expression plasmid. For cellular expression, A549 cells received either the recombinant construct or the empty vector using Lipofectamine LTX with Plus Reagent (Thermo Fisher Scientific, Waltham, MA, USA), following the supplier's recommended procedure. At 24 h post-transfection, selection was initiated by supplementing the culture with puromycin at a final concentration of 1 μg/mL. After two weeks of selection, individual colonies were isolated. Each clone was divided into two portions: one for Western blotting and the other for expansion and cryopreservation.

CRISPR/Cas9-based gene disruption of S100A4 was performed using guide sequences designed through the CHOPCHOP web-based platform (https://chopchop.cbu.uib.no/). The sgRNA oligonucleotide sequences include forward sense, CACCGGTGCCCTCTGGAGAAGGCCC; reverse sense, AAACGGGCCTTCTCCAGAGGGCACC. The complementary oligonucleotides were subjected to phosphorylation and annealing before being inserted into the BbsI-digested pX459 vector (Addgene) by ligation. Correct assembly of the resulting pX459-S100A4-sgRNA plasmid was confirmed by Sanger sequencing. The validated construct was subsequently introduced into A549 cells following the transfection procedure described above. At 24 h post-transfection, cells were distributed into 96-well plates, with an average seeding density of 0.5 cell per well. After two weeks of culture, single-cell clones were transferred to 24-well plates, and each clone split into two aliquots. After an additional ten days, one aliquot was analyzed ty sequencing and Western blotting analysis to confirm loss of S100A4 protein expression, while the corresponding aliquot was expanded for cell stock preparation.

### 2.4 Co-immunoprecipitation and Western blotting analysis

To achieve higher protein expression, the M2 gene derived from the WSN virus was codon-optimized for human expression and chemically synthesized by GenScript. The resulting construct, pCAGGS-M2-Flag, was generated by inserting a humanized M2 coding sequence with an N-terminal Flag tag into the pCAGGS vector. For S100A4, the coding sequence of the human gene was obtained by PCR using A549 cell cDNA as a template and subsequently ligated into pCAGGS to generate pCAGGS-S100A4-Myc, which carries a C-terminal Myc tag. A series of M2 truncation mutants (aa 26–97, Δ45–62, 1–70, 1–90), each bearing an N-terminal Flag tag, were also generated by cloning M2 fragments of varying lengths into pCAGGS. All recombinant plasmids were verified by Sanger sequencing to exclude unintended mutations. Primer sequences can be provided upon request.

293T cells were cultured in 6-well plates and subsequently subjected to co-transfection with the indicated plasmids according to a previously reported method (Feng et al., 2022a). At 48 h post-incubation, cellular material was collected after two washes with ice-cold phosphate-buffered saline. Cell lysis was then performed using IP lysis buffer composed of 50 mM Tris-HCl (pH 7.4), 150 mM NaCl, 1 mM EDTA, 1% NP-40, and 5% glycerol, with a protease inhibitor cocktail included to prevent proteolytic degradation. Lysis proceeded on ice for 30 min with intermittent vortexing. Insoluble material was pelleted by centrifugation at 12,000 g for 10 min at 4 °C. The clarified supernatants, containing equal amounts of total protein for each sample, were first pre-incubated with 20 μL of Protein A/G agarose beads (Yeasen Biotechnology, Shanghai, China) to reduce nonspecific binding. Subsequently, lysates were incubated overnight at 4 °C with the specified primary antibody, after which Protein A/G agarose was added, and the mixtures were rotated gently for 6–12 h at 4 °C. Beads were harvested, followed by four washes in IP buffer. The immunoprecipitated proteins were then separated via SDS-PAGE and blotted onto polyvinylidene fluoride (PVDF) membranes, which were probed with the specified antibodies. The resulting chemiluminescence was detected and recorded using the Tanon 5200 Chemiluminescent Imaging System (Tanon, Shanghai, China).

### 2.5 Antibodies

The antibody panel consisted of commercially available and laboratory-generated reagents. The primary antibodies comprised anti-S100A4 (rabbit monoclonal, Cell Signaling Technology, 13018S; mouse monoclonal, produced in our laboratory), anti-β-actin (mouse, Proteintech, 66009-1-Ig), anti-Flag (mouse, Sigma-Aldrich, F1084), anti-Myc (rabbit polyclonal, Abcam, ab9106), and anti-M2 (mouse monoclonal, produced in our laboratory). Secondary antibody detection was performed with HRP-conjugated goat anti-rabbit IgG (Beyotime, A0208) or HRP-conjugated goat anti-mouse IgG (Beyotime, A0216).

### 2.6 Viral growth kinetics

For infection experiments, to allow multiple rounds of viral replication, six-well plates containing WT and S100A4-deficient A549 cells were infected with WSN virus at a multiplicity of infection (MOI) of 0.01. For infection, cells were maintained in Ham's F12K medium supplemented with 0.3% BSA and 0.4 μg/mL TPCK-treated trypsin. All infected cultures were kept at 37 °C. At 48 h post-infection, the culture medium was collected, and infectious virus levels were subsequently determined using a plaque-forming assay in MDCK cells.

### 2.7 Immunofluorescence

For immunofluorescence analysis, to ensure synchronous and efficient infection of the majority of cells at an early time point and provide sufficient viral M2 protein signal for reliable co-localization analysis, A549 cells were grown in glass-bottom dishes and exposed to the WSN virus at an MOI of 10. Cells were collected at the indicated post-infection time points, washed with ice-cold PBS, and fixed with 4% paraformaldehyde for 30 min at room temperature or overnight at 4 °C. Following two additional PBS washes, membrane permeabilization was performed with 0.1% Triton X-100 for 15 min at room temperature. The cells were then blocked with Blocking One for 1 h at room temperature, after which the primary antibodies diluted in Blocking One (Nacalai Tesque, Kyoto, Japan) were applied and allowed to incubate overnight at 4 °C. After four washes, secondary antibodies bearing Alexa Fluor 488 or Alexa Fluor 594 were obtained from Thermo Fisher Scientific (Waltham, MA, USA).and applied for fluorescence detection. For subsequent staining, the antibody solution consisted of PBS-T supplemented with 20% Blocking One and DAPI (Thermo Fisher Scientific, Waltham, MA, USA), and the samples were maintained at room temperature for 60 min. Subsequently, the cells were washed four times and visualized using a CSIM130 Confocal Microscope (Sunny Technology, Beijing, China).

### 2.8 Rluc reporter assay with WSN PB2-knockout virus

To obtain efficient cellular infection while restricting the analysis to a single round of viral replication, A549 cells with or without S100A4 were distributed into 24-well plates and infected the following day with WSN PB2-KO/Rluc at an MOI of 1. Amantadine at 100 μg/mL (Sigma-Aldrich) was included as a positive control. The virus was allowed to interact with the cells for 1 h, after which the inoculum was removed and the wells were replenished with 0.3% BSA-containing Ham's F12K medium supplemented either with or without amantadine. Eight h after infection, the medium was aspirated and the cells were subjected to measurement of *Renilla* luciferase activity using the Promega *Renilla* Luciferase Assay System and a SpectraMax^®^ i3x Multi-Mode Microplate Reader (Molecular Devices), following the respective manufacturer's protocol.

### 2.9 Minigenome assay

A549 cells of either the WT or S100A4-deficient genotype were cultured in 24-well plates before transfection. One day later, each well received a plasmid mixture containing the four WSN polymerase-related expression constructs (PB2, PB1, PA, and NP), the firefly luciferase-encoding viral-like RNA construct pPolINP(0)luc2(0), and pNull-Rluc for internal normalization. This assay was performed according to the previously published procedure (Feng et al., 2022b) and the transfection conditions described above. Cell lysates collected 24 h thereafter were analyzed for firefly and *Renilla* luciferase activity using the Dual-Glo Luciferase Assay System (Promega, E2920) and a SpectraMax^®^ i3x Multi-Mode Microplate Reader (Molecular Devices).

### 2.10 IFN-β ELISA

For IFN-β determination, WT and S100A4-overexpressing A549 cells were infected with PR8-NS1-R38A/K41A at an MOI of 1. Following incubation at 37 °C for 24 h, supernatants were collected for cytokine analysis. IFN-β concentrations were assessed with the Human Interferon Beta (IFN-β) ELISA Kit (Elabscience, Wuhan, China), following the supplier's recommended procedure, and absorbance values were recorded at 450 nm using an AMR-100 microplate reader (Allsheng, Hangzhou, China). For qRT-PCR quantification of ISG15 and BST2 transcript expression, total RNA was isolated and reverse-transcribed into cDNA using the specified protocols. Amplification was performed with the designated primer sets under the established cycling conditions, and GAPDH served as the endogenous reference for normalization of ISG15 and BST2 mRNA expression levels.

### 2.11 Statistical analysis

All statistical calculations were carried out with GraphPad Prism version 6.07 (GraphPad Software, Inc., San Diego, CA, USA). Experimental groups were compared by one-way or two-way ANOVA, as appropriate, with Dunnett's test used for subsequent multiple comparisons. Statistical significance was defined as *p* < 0.05.

## 3 Results

### 3.1 S100A4 is essential for the propagation of influenza A virus in A549 cells

The potential role of S100A4 in IAV replication was evaluated in A549 cells using an RNAi approach. Three S100A4-specific siRNAs were introduced into the cells, followed by verification of S100A4 depletion through quantitative RT-PCR and Western blot analysis (Figures 1A, B). Knockdown of S100A4 had no appreciable effect on cellular viability, as the viability of cells treated with S100A4-targeting siRNAs was similar to that of cells receiving the negative-control siRNA (siNC) (Figure 1C). To examine the role of S100A4 during viral infection, cells transfected with either S100A4-specific siRNAs or control siRNAs were challenged with WSN virus at an MOI of 0.01. Viral production was assessed 48 h after infection and was substantially lower in S100A4-depleted cells than in the corresponding siNC controls (Figure 1D). These findings indicate that S100A4 is required for efficient completion of the influenza virus replication cycle. To validate the role of S100A4 in influenza virus replication in A549 cells, we generated an S100A4-deficient A549 cell line using CRISPR/Cas9 genome editing. Briefly, WT A549 cells were introduced with the pX459 construct encoding a guide RNA targeting exon 2 of the human S100A4 locus. Following single-cell cloning, genomic sequencing identified a one-nucleotide thymidine deletion within exon 2 in clone 4, resulting in a frameshift mutation (Figure 2A). Consistent with the genomic alteration, Western blot analysis with an S100A4-specific antibody showed that S100A4 protein was undetectable in this clone (Figure 2B). Importantly, disruption of S100A4 did not significantly affect cell viability, which remained comparable between the knockout and parental WT cells 24 h after seeding (Figure 2C).

**Figure 1:**
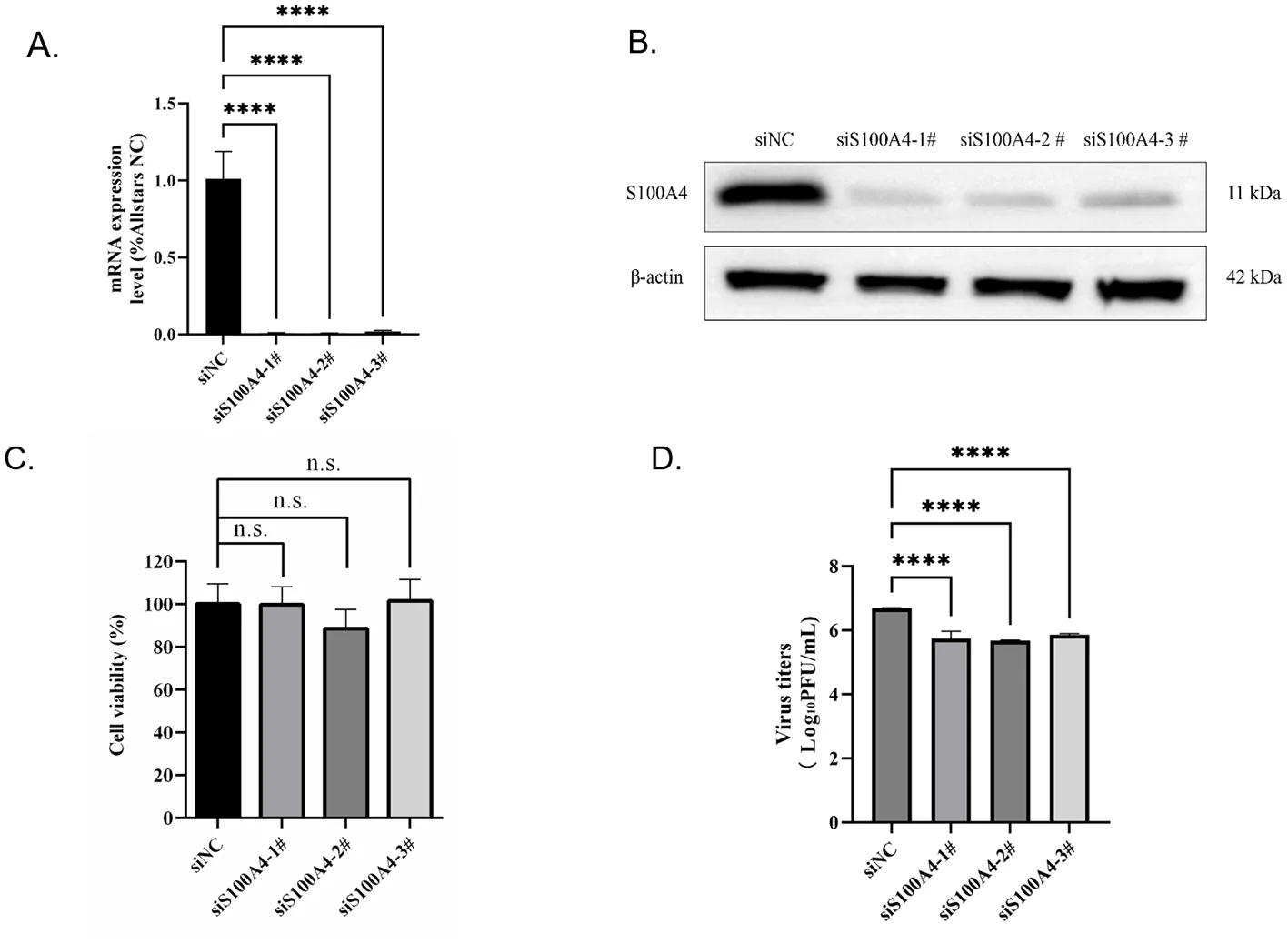
Silencing of S100A4 led to decreased influenza virus replication. Two sequential transfections were performed in A549 cells using negative control siRNA (siNC) or three individual siRNAs directed against human S100A4 (siS100A4-1#, siS100A4-2#, siS100A4-3#). **(A)** The S100A4 mRNA amount in siRNA-treated A549 cells was determined by qPCR, confirming the reduced expression of S100A4 mRNA. **(B)** The extent of S100A4 depletion was evaluated by Western blotting using antibodies against S100A4 and β-actin. **(C)** The viability of A549 cells following siRNA treatment was examined using the CCK-8 assay. **(D)** siRNA-transfected A549 cells were infected with WSN virus at an MOI of 0.01, and viral replication was evaluated by analyzing culture supernatants collected at 48 h post-infection. Results in **(A)**, **(C)**, and **(D)** are shown as mean ± SD from three independent experiments. Statistical significance was determined using two-way ANOVA with Dunnett's multiple-comparison test; ****p < 0.0001. n.s. indicates not significant.

**Figure 2:**
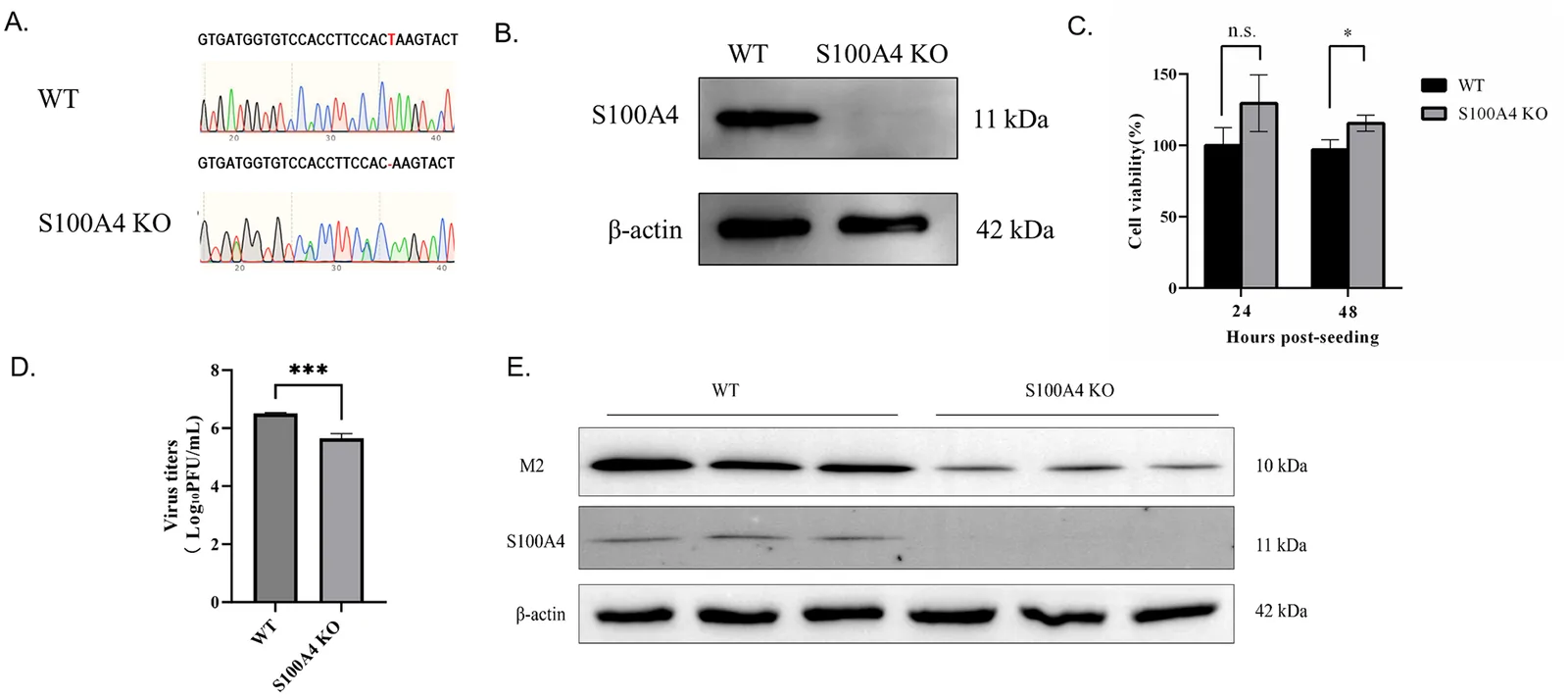
Knockout of S100A4 in A549 cells markedly attenuated influenza A virus propagation. **(A)** A single thymine nucleotide deletion at exon 2 of the S100A4 gene was verified by Sanger sequencing. **(B)** Western blot analysis confirmed the lack of S100A4 expression in A549 cells. **(C)** Cell viability of S100A4 KO cells was assessed using the CCK-8 assay. **(D)** S100A4 deletion led to a significant decrease in WSN virus growth. **(E)** Loss of S100A4 caused a marked decline in M2 expression following viral infection. * *p* < 0.05, ***p < 0.001 (two-way ANOVA followed by Dunnett's test). n.s. indicates not significant.

We next examined whether S100A4 deficiency altered the kinetics of WSN virus replication using a multicycle growth experiment. WT and S100A4-KO A549 cells were infected with WSN virus at an MOI of 0.01, and culture supernatants were harvested at the indicated time points. Infectious virus yields were subsequently quantified by plaque assay. Compared with WT cells, S100A4-KO cells produced significantly lower levels of infectious WSN virus and exhibited reduced accumulation of the viral M2 protein (Figures 2D, E). Together, these data provide genetic evidence that S100A4 facilitates efficient influenza virus replication in A549 cells.

### 3.2 Elevated S100A4 expression enhances influenza A virus propagation in A549 cells

To further validate the aforementioned results, A549 cell lines stably overexpressing S100A4, along with vector controls, were established. Western blotting analysis confirmed that S100A4 protein levels were elevated in these cells compared with vector-transfected controls (Figure 3A). At 48 h post-infection, viral titers and M2 expression levels in S100A4-overexpressing A549 cells exceeded those in vector-control cells, whereas cell viability remained comparable between the two groups (Figures 3B–D). These observations indicate that S100A4 functions as a host factor that facilitates influenza A virus infection in A549 cells.

**Figure 3:**
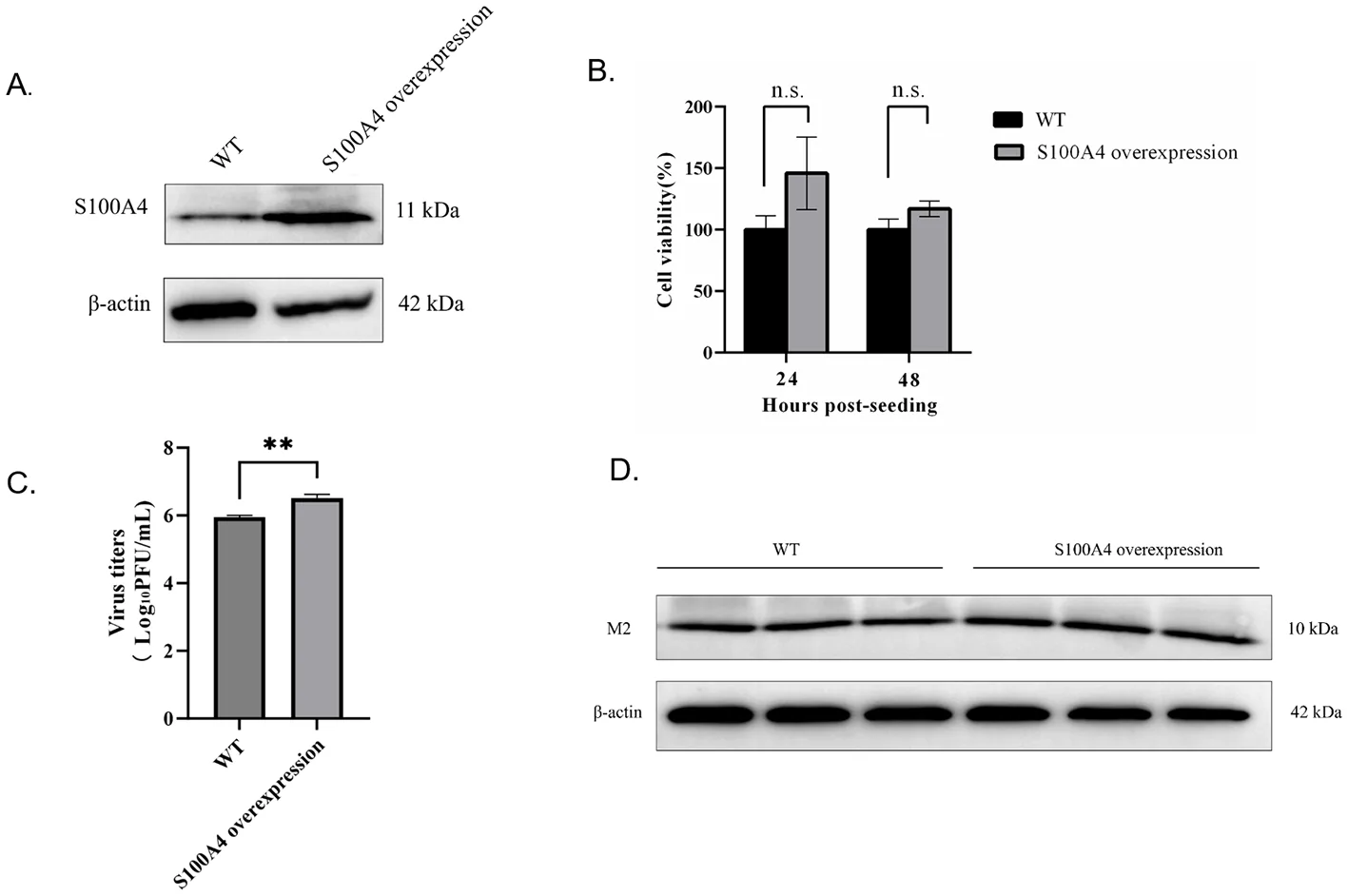
Elevated expression of S100A4 in A549 cells promoted influenza A virus replication. **(A)** S100A4 overexpression in A549 cells was verified using an anti-S100A4 antibody. **(B)** Cell viability of S100A4-overexpressing A549 cells was measured by the CCK-8 assay. The cell viability was comparable between the two groups at 24 h and 48h post-incubation, respectively. **(C)** S100A4 overexpression in A549 cells increased WSN virus growth. **(D)** S100A4 overexpression in A549 cells upregulated the expression level of the M2 protein. **p < 0.01 (two-way ANOVA followed by Dunnett's test). n.s. indicates not significant.

### 3.3 S100A4 interacts with the influenza A virus M2 protein via residues 45–62 of M2

To investigate whether M2 associates with S100A4, we employed a co-immunoprecipitation approach in HEK293T cells, which are well suited for transient protein expression owing to their high transfection efficiency. Cells were simultaneously transfected with pCAGGS-M2-Flag, expressing Flag-tagged M2, and pCAGGS-S100A4-Myc, expressing Myc-tagged S100A4. Cell extracts were collected 48 h after transfection and subjected to immunoprecipitation using either anti-Flag or anti-Myc antibodies. The resulting immune complexes were analyzed by Western blotting to detect M2 and S100A4. Notably, S100A4 was detected in the M2 immunoprecipitate, whereas M2 was also recovered from the S100A4 immunoprecipitate, demonstrating a reciprocal association between the two proteins (Figure 4A). The intracellular distribution of S100A4 during IAV infection was examined by monitoring its spatial relationship with M2. A549 cells were exposed to WSN virus at an MOI of 10 and fixed at 8 h post-infection. Immunofluorescence analysis showed that S100A4 was detectable in both the cytoplasmic and nuclear compartments in mock- and virus-infected cells, with a predominant cytoplasmic localization. In WSN-infected cells, the cytoplasmic S100A4 signal overlapped with that of M2 (Figure 4B). Together, these results indicate that IAV infection has little effect on the intracellular localization of S100A4 and support the notion that S100A4 associates with M2 during viral infection.

**Figure 4:**
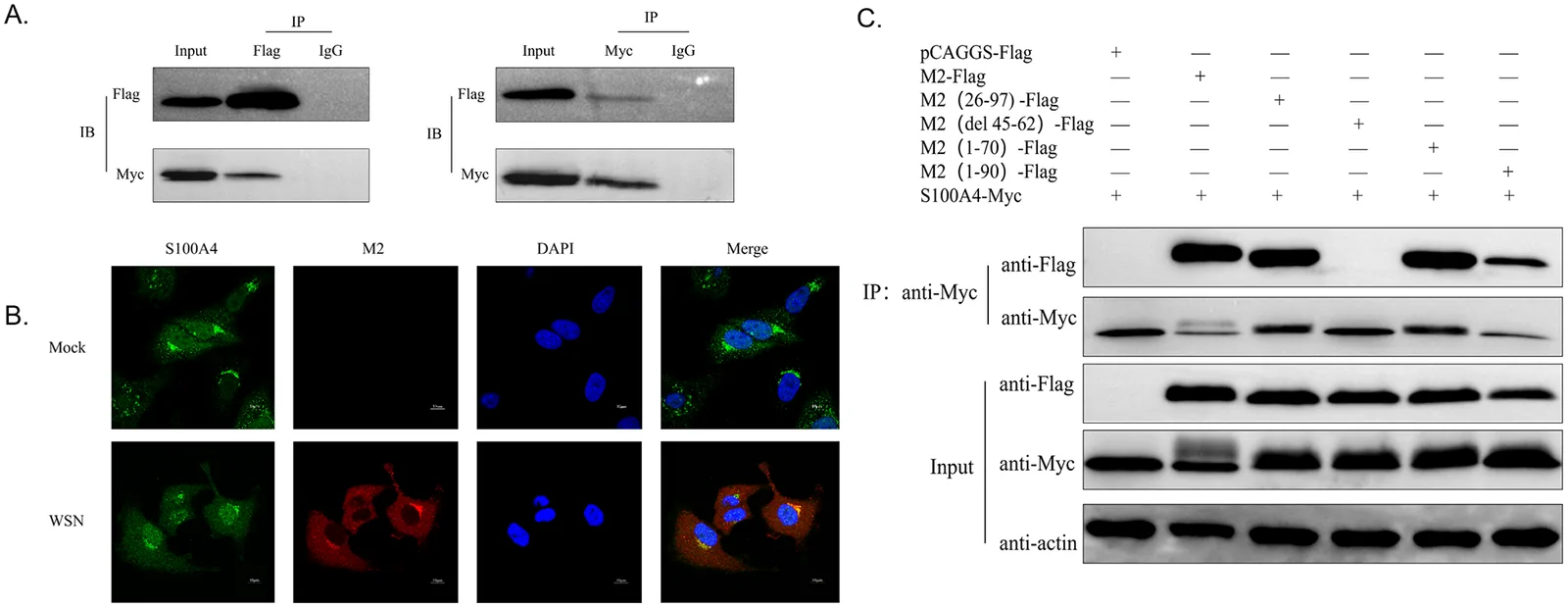
S100A4 interacted with M2 proteins of influenza A virus mediated by residues 45-62 of M2 protein. **(A)** Association of M2 with S100A4 in human cells. HEK293T cells were co-transfected with plasmids encoding Flag-tagged M2 (pCAGGS-M2-Flag) and Myc-tagged S100A4 (pCAGGS-S100A4-Myc). Cell extracts were subsequently collected and analyzed by reciprocal co-immunoprecipitation. Protein complexes were isolated using antibodies against either Flag or Myc, followed by immunoblot analysis with the corresponding reciprocal antibody to assess the presence of M2 and S100A4 in the immunoprecipitated complexes. **(B)** A549 cells were infected with WSN virus at an MOI of 10. Following an 8-h incubation period, the cells were fixed and permeabilized before being subjected to dual immunofluorescence staining using antibodies specific for M2 and S100A4. The intracellular distribution of these proteins was subsequently examined by confocal laser-scanning microscopy. **(C)** Identification of the M2 domain required for association with S100A4. The indicated Flag-fused M2 deletion variants were co-expressed with S100A4-Myc in 293T cells. Protein complexes recovered by anti-Flag or anti-Myc immunoprecipitation were examined by reciprocal Western blotting. The Input panels serve as controls for protein expression and loading, and the critical observation is the absence of interaction for the Δ45-62 mutant.

To delineate the region of M2 that mediates its association with S100A4, a series of M2 deletion constructs were individually expressed in 293T cells, followed by co-immunoprecipitation analysis to assess their ability to associate with S100A4. As shown in Figure 4C, removal of the segment encompassing residues 45–62 completely disrupted the detectable association between M2 and S100A4. Collectively, these findings identify the 45–62 amino acid region of M2 as a critical determinant required for its interaction with S100A4.

### 3.4 S100A4 participates in the early stages of influenza virus replication

To determine whether S100A4 participates in the early stages of influenza virus replication, we used the PB2-deficient, replication-incompetent WSN reporter virus carrying *Renilla* luciferase (WSN PB2-KO/Rluc). WT and S100A4 KO A549 cells received the virus at an MOI of 1 to obtain efficient cellular infection while restricting analysis to a single round of viral replication. Amantadine served as the positive-control treatment, based on its established inhibition of the M2 proton channel and viral uncoating process (Wharton et al., 1994). Reporter activity was subsequently determined by measuring *Renilla* luciferase at 8 h post-infection. Both S100A4 deficiency and amantadine treatment led to a marked decrease in *Renilla* luciferase activity (Figure 5A). In S100A4 KO A549 cells, *Renilla* luciferase activity was diminished by 40–50% relative to WT A549 cells. These findings indicate that S100A4 acts as a positive regulator of the early events in the influenza A virus life cycle.

**Figure 5:**
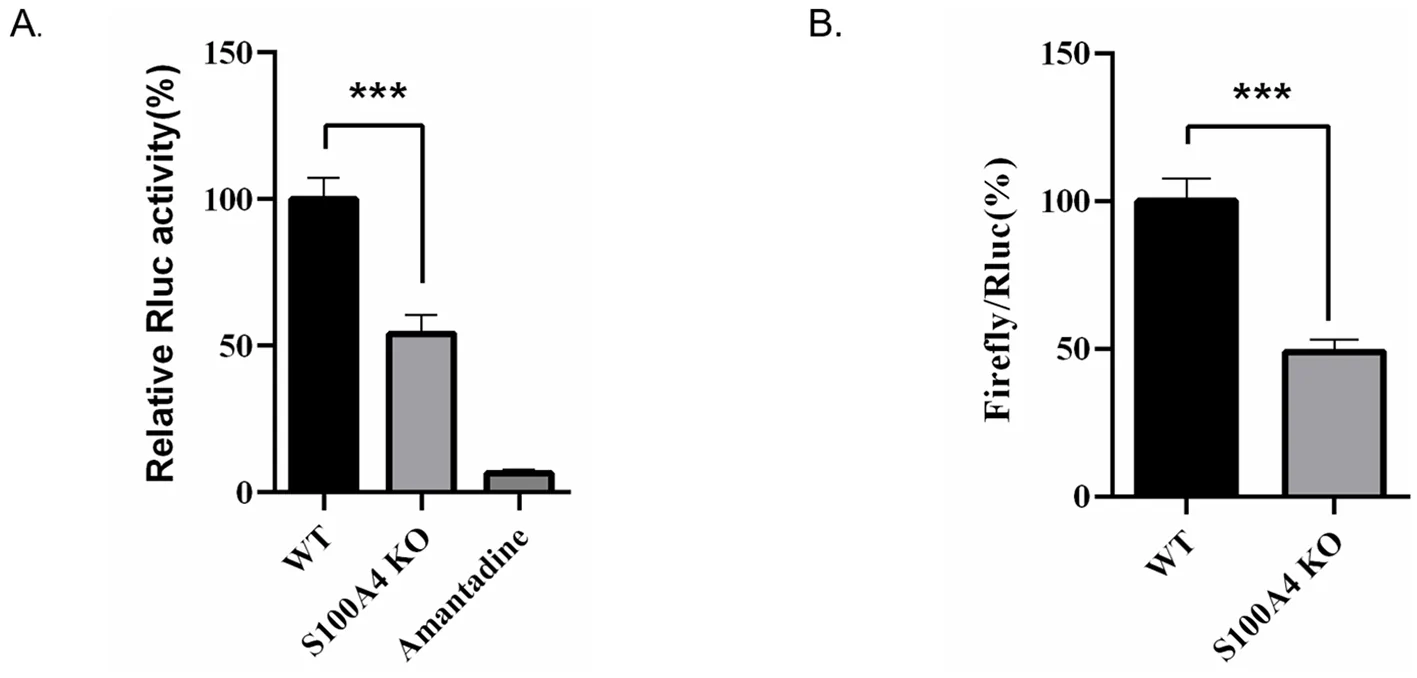
Role of S100A4 at different stages of the influenza virus life cycle. **(A)** S100A4 affects early-stage IAV replication. A549 cells with intact or disrupted S100A4 expression were infected with WSN PB2-KO/Rluc at an MOI of 1 in the presence of the positive-control compound amantadine. *Renilla* luciferase activity was assessed 8 h after infection. Values are presented as mean ± SD (n = 3 independent experiments). Statistical significance was evaluated by one-way ANOVA with Dunnett's *post hoc* analysis; ***p < 0.001. **(B)** Effect of S100A4 deletion on IAV mini-genome activity. The viral polymerase components PB1, PB2, PA, and NP from WSN were expressed together with firefly and *Renilla* luciferase reporter constructs in WT or S100A4-KO A549 cells. Luciferase activities were measured using the Dual-Glo system. Results are shown as mean ± SD (three replicates), with WT A549 cells normalized to 100%. ***p < 0.001 versus the WT control.

### 3.5 Depletion of S100A4 attenuates influenza A virus polymerase activity

To determine whether the absence of S100A4 affects the polymerase activity of influenza A virus, A549 cells with either intact or disrupted S100A4 expression were subjected to plasmid-based reconstitution of the viral polymerase complex. The transfection mixture contained expression vectors for PB2, PB1, PA, and NP, along with the firefly luciferase reporter pPolINP(0)luc2(0) and the *Renilla* luciferase control pNull-Rluc. Luciferase reporter signals from both enzymes were subsequently quantified, and their ratio was used to assess polymerase activity. We observed that S100A4 depletion led to a substantial decrease in polymerase activity (Figure 5B). These data suggest that S100A4 is necessary for sustaining the polymerase activity of influenza virus in A549 cells.

### 3.6 S100A4 suppresses interferon production following influenza virus infection

To determine whether S100A4 is involved in interferon (IFN) production during influenza virus replication, vector-control and S100A4-overexpressing A549 cells were infected with the A/Puerto Rico/8/1934(H1N1) -NS1-R38A/K41A strain at an MOI of 1, which induces interferon production (Donelan et al., 2003). IFN-β levels in the culture supernatant were quantified at 24 h post-infection. The results revealed that IFN-β levels were significantly reduced in the supernatants of infected S100A4-overexpressing A549 cells (Figure 6A). Moreover, the mRNA levels of the interferon-stimulated genes (ISGs) ISG15 and BST2 were markedly reduced in the S100A4-overexpressing cells compared to those in vector-control cells (Figures 6B, C). These findings indicate that S100A4 suppresses interferon production during influenza A virus infection.

**Figure 6:**
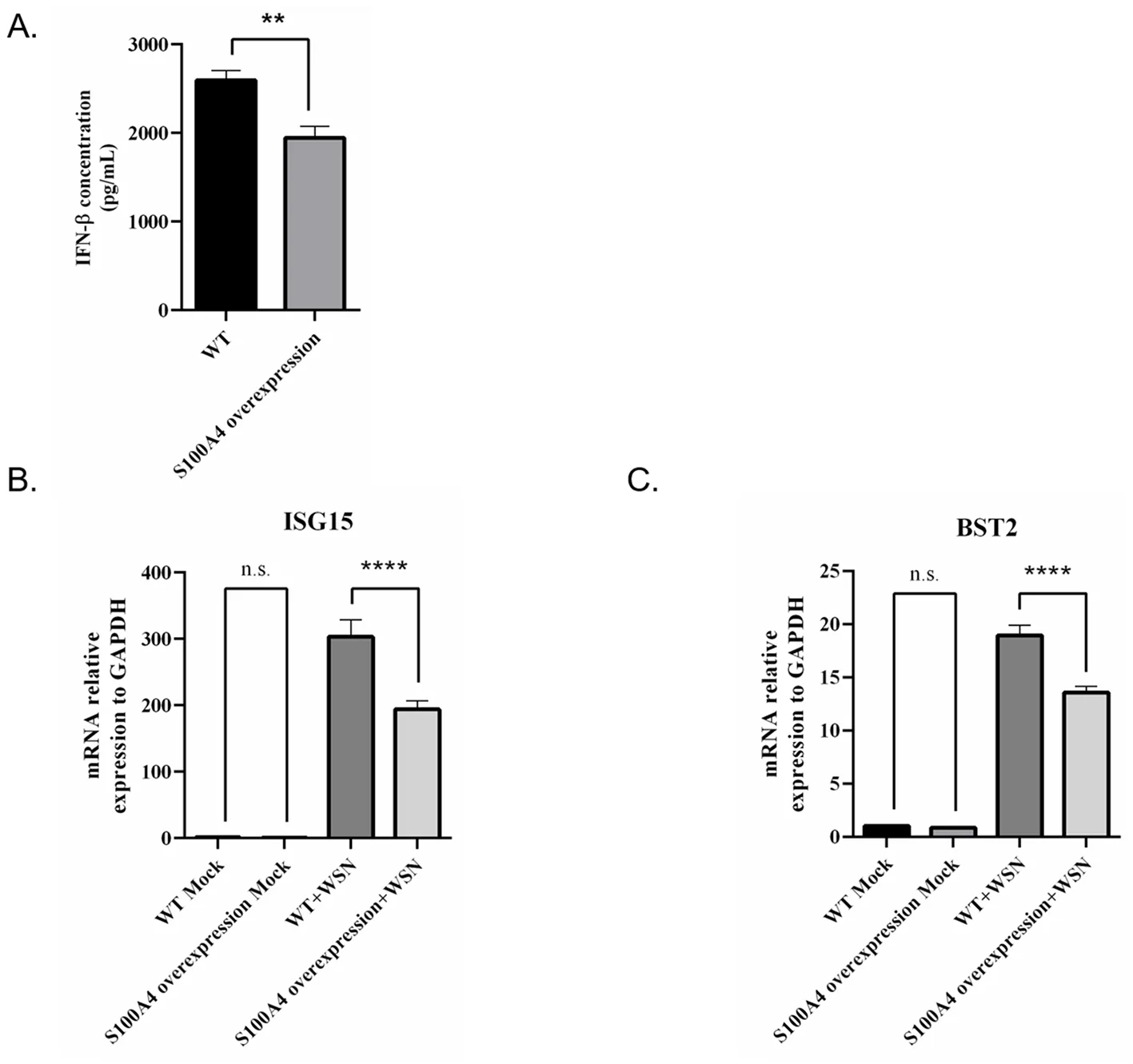
Effect of S100A4 on influenza virus-induced IFN-β production. **(A)** WT and S100A4-overexpressing A549 cells were infected with PR8-NS1-R38A/K41A virus at an MOI of 1 and incubated at 37 °C for 24 h post-infection. Supernatants were harvested, and IFN-β levels were quantified using a Human IFN-β ELISA Kit. **(B)** qRT-PCR revealed that ISG15 was downregulated following elevated S100A4 expression. **(C)** qRT-PCR revealed that BST2 was downregulated following elevated S100A4 expression. **p < 0.01, ****p < 0.0001 (one-way ANOVA followed by Dunnett's test).

## 4 Discussion

The interplay between viral and host factors is essential for the influenza virus life cycle. Our findings reveal a previously uncharacterized role for S100A4 in the IAV life cycle. In A549 cells, genetic depletion and enhanced expression of S100A4 produced opposing effects on viral replication, supporting S100A4 as a host factor that positively regulates IAV propagation. In addition, S100A4 was identified as a cellular protein associated with the viral M2 protein. The role of S100A4 in viral replication appears virus-specific. In Zika virus (ZIKV), S100A4 primarily facilitates long-term viral persistence in an immune-privileged site, the testis, through an indirect, immune cell-mediated mechanism (Yang et al., 2024); whereas in influenza A virus, it acts as a direct proviral host factor that participates in multiple key steps of the replication cycle and suppresses the interferon response (Figures 5, 6). This dual function positions S100A4 as a notable node in virus–host interactions, although the precise molecular mechanisms underlying its actions across different viruses remain to be further elucidated.

The cytoplasmic tail of M2 contains an amphipathic α-helix within residues 45–62. This region has been demonstrated to mediate membrane curvature induction and ESCRT-independent membrane scission during virus budding (Rossman et al., 2010). Structural analyses revealed that this helix folds upon membrane binding, with its hydrophobic face inserting into the lipid bilayer, thereby driving membrane binding through hydrophobic rather than electrostatic interactions, and consequently inducing membrane curvature and lipid ordering to complete ESCRT-independent membrane scission (Rossman et al., 2010; Martyna et al., 2017). The 45–62 region may interact with S100A4 because its amphipathic helical structure provides a physical interface. The helix possesses both hydrophobic and hydrophilic surfaces, offering a recognizable binding platform for S100A4. Given that S100A4 itself functions in membrane and cytoskeletal regulation, the two proteins are functionally complementary and may be co-opted by M2 as an auxiliary factor for membrane remodeling.

As early as 1985, the influenza virus M2 protein was shown to localize to the Golgi apparatus (Zebedee et al., 1985), and subsequent studies further clarified its critical role within the trans-Golgi network (TGN) (Sze and Tan, 2015). The core function of M2 in the TGN is to utilize its proton channel activity to maintain the pH of this compartment, thereby preventing the premature acid-induced conformational change of newly synthesized hemagglutinin (HA) during transport (Pinto and Lamb, 2006; Sze and Tan, 2015). S100A4, a calcium-binding protein, localizes predominantly in the cytoplasm and nucleus, and is secreted extracellularly via an endoplasmic reticulum–Golgi-dependent pathway (Herwig et al., 2016), a trafficking route that passes through the TGN. Moreover, S100A4 has been suggested to co-localize with the Golgi apparatus (Tao et al., 2026). The co-localization of M2 and S100A4 in the TGN could provide a spatial context for the virus to exploit host membrane/cytoskeletal regulatory factors to facilitate its membrane trafficking and budding processes (Figure 4B). The amphipathic helix formed by residues 45–62 has been demonstrated to be the key structural domain mediating membrane curvature induction and virus budding (Figure 4C) (Pinto and Lamb, 2006), whereas S100A4 possesses actin-binding and membrane- dynamics regulatory capabilities (Tao et al., 2026). These two proteins are therefore functionally highly complementary. We speculate that M2 may recruit S100A4 as an auxiliary factor for membrane remodeling within the TGN through its 45–62 region, thereby optimizing the efficiency of viral assembly and release.

The influenza virus M2 protein facilitates viral uncoating through its proton channel activity, thereby releasing vRNPs into the cytoplasm for transport to the nucleus, where transcription and replication are initiated (Ren et al., 2026). Additionally, M2 stabilizes the viral polymerase by blocking autophagosome-lysosome fusion and activating AKT-mTOR signaling, thereby indirectly sustaining polymerase activity (Gannage et al., 2009). S100A4 may not directly participate in polymerase catalysis; however, it may facilitate the intracellular trafficking of vRNPs by regulating cytoskeletal dynamics and may alleviate interferon-mediated antiviral suppression of the polymerase by inhibiting interferon production (Elliott et al., 2012). The interaction between M2 and S100A4 may sustain polymerase activity through two complementary mechanisms (Figure 5B). On one hand, M2-mediated autophagy inhibition and polymerase stabilization; on the other hand, S100A4-mediated cytoskeletal regulation that optimizes vRNP transport to the nucleus. Together, these two proteins may functionally complement each other to create a favorable environment for viral polymerase activity.

The M2 protein regulates innate immunity through both channel activity-dependent and -independent mechanisms. On one hand, M2 anchors to mitochondria and directly interacts with MAVS, promoting MAVS aggregation by inducing ROS production while antagonizing autophagy to prevent the degradation of MAVS aggregates, thereby enhancing RLR signaling (Wang et al., 2019). On the other hand, the channel activity of M2 triggers the release of mitochondrial DNA into the cytoplasm, which induces IFN-β expression via the cGAS/DDX41–STING pathway, although the NS1 protein can bind mtDNA to help the virus evade this DNA-sensing pathway (Moriyama et al., 2019). S100A4 also modulates interferon responses through direct binding. S100A4 binds IFN-β in a calcium-dependent manner and suppresses IFN-β-induced cellular effects (Kazakov et al., 2020). Upon interaction between M2 and S100A4, the combined effects of M2-mediated regulation of MAVS aggregation and S100A4-mediated direct binding of IFN-β may synergistically suppress antiviral output of innate immunity, thereby indirectly sustaining an environment favorable for polymerase activity (Figures 5B, 6A).

Overall, the present study supports a functional role for S100A4 in facilitating influenza A virus infection of human lung epithelial cells. Rather than acting at a single step, S100A4 appears to affect multiple processes associated with the viral replication program, including the establishment of productive infection, accumulation of viral proteins, and preservation of viral RNA polymerase function. We further demonstrate that S100A4 is capable of attenuating type I interferon induction, indicating that this host protein may contribute to the regulation of innate antiviral defenses during infection. Further investigation of the molecular interplay between S100A4 and influenza A virus may expand our understanding of the mechanisms governing viral replication and, in the longer term, inform the development of host-directed antiviral strategies, although the safety and selectivity of targeting S100A4 remain to be evaluated.

## Data Availability

The raw data supporting the conclusions of this article will be made available by the authors, without undue reservation.
